# Optimization to the Culture Conditions for* Phellinus* Production with Regression Analysis and Gene-Set Based Genetic Algorithm

**DOI:** 10.1155/2016/1358142

**Published:** 2016-08-16

**Authors:** Zhongwei Li, Yuezhen Xin, Xun Wang, Beibei Sun, Shengyu Xia, Hui Li, Hu Zhu

**Affiliations:** ^1^College of Computer and Communication Engineering, China University of Petroleum, Qingdao, Shandong 266580, China; ^2^Center for Bioengineering and Biotechnology, China University of Petroleum, Qingdao, Shandong 266580, China

## Abstract

*Phellinus* is a kind of fungus and is known as one of the elemental components in drugs to avoid cancers. With the purpose of finding optimized culture conditions for* Phellinus* production in the laboratory, plenty of experiments focusing on single factor were operated and large scale of experimental data were generated. In this work, we use the data collected from experiments for regression analysis, and then a mathematical model of predicting* Phellinus* production is achieved. Subsequently, a gene-set based genetic algorithm is developed to optimize the values of parameters involved in culture conditions, including inoculum size, PH value, initial liquid volume, temperature, seed age, fermentation time, and rotation speed. These optimized values of the parameters have accordance with biological experimental results, which indicate that our method has a good predictability for culture conditions optimization.

## 1. Introduction

*Phellinus* is a kind of fungus having great medicinal value, since it is known as one of the elemental components in drugs with functions of avoiding cancers [1, 2].* Phellinus* flavonoids are one of the most popular parasitifers of* Phellinus* in nature [3], and the research on* Phellinus* focuses on polysaccharides, proteoglycans medicinal mechanism, composition, and so forth, which are mostly extracted from the fruiting bodies of* Phellinus* flavonoids [4].* Phellinus* rarely exists in the wild environment [5], and it becomes a promising research branch to cultivate it in the laboratory. With mycelial growth by liquid fermentation, the fermentation broth flavonoids, polysaccharides, alkaloids, and other active substances can be produced, which have high level physical activity, short fermentation period, and mass productions, thus providing a possible way of producing* Phellinus* in the laboratory [6]. In recent years, updated machine learning approaches (see, e.g., [7, 8]) have been developed and applied in biological data processing.

From the understanding of the wild conditions of* Phellinus*, it is believed that PH value, temperature, and fermentation time have effect on the productions. Also, in general biochemical experiments, we need to consider the inoculum size, initial liquid volume, seed age, and rotation speed. In the laboratory, plenty of experiments have been designed and operated for maximizing the* Phellinus* production. The methods can be separated into two major groups.

- With biological technologies: it used optimum media on mycelial growth of* Phellinus* in [9] and liquid fermentation technology to cultivate* Phellinus* in [10]. Active ingredients in* Phellinus* and polysaccharide metabolism regulation are designed in [11].
- With mathematical models: some researches focus on building mathematical models for the progress of producing* Phellinus* by differential equations [12], metabolic path and network [13], and complex network models [14].

Artificial algorithms and models have been used in the bioprocess, particularly for the optimization of culture conditions. In [15], artificial neural network (ANN) is used to optimize the extraction process of azalea flavonoids. Neural networks combined with evolutionary algorithms have been used to optimize the experimental environment, such that neural network and particle swarm optimization method were used for finding optimized culture conditions to maximize the production of Pleuromutilin from* Pleurotus mutilus* in [16]. Recently, with the increment of biological data, regression analysis becomes a useful tool for the data analysis. In [17] the method of fitting models to biological data using linear and nonlinear regression is proposed, where some multivariate statistical analysis strategies from [18, 19] are formulated to be helpful and useful for biologists. These results give us hints of using regression analysis and artificial algorithms to optimize the culture conditions for* Phellinus* production. And, to the best of our knowledge, few work focuses on the optimization of culture conditions to maximize the production of* Phellinus* in the laboratory.

In this work, we start from operating 45 experiments for producing* Phellinus* from* Phellinus* flavonoids with different culture conditions, involving parameters PH value, temperature and fermentation time, inoculum size, initial liquid volume, seed age, and rotation speed. With the data collected during the experiments, we use regression analysis method to create a mathematical model, which can forecast the flavonoid yield and the most important element to the production of* Phellinus*. After that, a gene-set based genetic algorithm (GA) is proposed to optimize the culture condition, where the obtained mathematic model is used as fitness function for the evolution of individuals. Data experimental results show that predicted optimal values of the parameters have accordance with biological experimental results, which indicate that our method has a good predictability for culture conditions optimization.

## 2. Data Collected from Experiments

In this section, biological experiments are performed for finding optimal value of certain single factor.

In Table 1, experiments are operated for collecting data. In rows 1–14, it is associated with experiments with PH values ranging from 1 to 14, where the temperature is fixed to 28°C, initial volume is set to be 100 mL, the rotation speed is 140 r/m, and seed age is 8 days. Rows 15 to 20 are 6 experiments with initial volume ranging from 40 mL to 140 mL, where PH value is set to be 6, the best one obtained from experiments with PH values ranging from 1 to 14.

In Table 2, experiments with including inoculum ranging from 2% to 16% and temperature ranging from 25°C to 40°C are performed. In Table 3 the situations on experiments with fermentation time ranging from 1 to 12 hours are shown. From the in total 45 experiments, we collect data of culture conditions for production of* Phellinus*. Different culture conditions have a fundamental influence on the production of* Phellinus*, but the optimized culture conditions remain unknown.

## 3. Methods

We consider here using regression analysis and gene-set based genetic algorithm to find the optimized culture conditions for maximizing the production of* Phellinus*. In general, we convert the data collected in Section 2 to construct a mathematical model by regression analysis. And then, the obtained model can be used as fitness function for optimizing the culture condition with gene-set based genetic algorithm.

### 3.1. Regression Analysis

In statistical modeling, regression analysis is a statistical process for estimating the relationships among variables [20]. Regression analysis is one of the extremely versatile data analysis methods, which is appropriated to establish dependencies between variables based on observational data and widely used to analyze the data inherent law and to predict the result. Regression analysis can be divided into linear regression and nonlinear regression analysis [21], according to the type of relationship between the independent variables and dependent variables. In general, the relationship between variables is determined by the independent variables and dependent selected variables, by which regression models can be made. After that, it is used to solve the various parameters of the model based on the measured data and then evaluate whether the regression model can fit the observed data. If the model can fit the data well, then the model can be used to further predict based arguments [22]. The regression analysis is composed of the following steps [23, 24].

Regression analysis is widely used in data mining, particularly for biological data analysis in recent years, with the purpose of finding a feasible statistical law by the large amount of data of experiments. The general process is given as follows.

**Step 1 .**

Determine the variables.

**Step 2 .**

Establish the prediction model.

**Step 3 .**

Relate analysis.

**Step 4 .**

Calculate the prediction error.

**Step 5 .**

Determine the predicted value.

From the data collected in Section 2, it consists of seven independent variables and one dependent variable. The seven independent variables are inoculum size, PH values, initial liquid volume, temperature, seed age, fermentation time, and rotation speed. And, the dependent variable is flavonoid yield. From the observation of the experiments, it is found that some culture conditions are not suitable for production of* Phellinus*. These data are taken as extreme data are removed from regression analysis. Extreme data refers to the data which were measured in extreme experimental environment. Also duplicate data were cancelled. Only the following data are selected in regression analysis.

- Inoculum size 0.5%~1.2%.
- PH 5~7.
- Initial liquid volume 60~100 mL.
- Temperature 25~30°C.
- Seed age 4~9 days.
- Fermentation time 6~12 days.
- Rotation speed 140~200 r/m.

After data filtering, a statistical model is made to represent these data. It is known that there is a correlation between these data relationships, so we applied linear regression analysis to fit them. At this stage, a lot of models were tested one by one with IBM SPSS software and response surface methodology. The statistical model is *Y* = *A*1*∗X* ^2^(1)+⋯+*A*7*∗X* ^2^(7) + *B*1*∗X*(1)*∗X*(2) + *B*2*∗X*(1)*∗X*(3) + *B*3*∗X*(1) *∗* *X*(4) + *B*4*∗X*(1)*∗X*(5) + *B*5*∗X*(1)*∗X*(6) + *B*7*∗X*(1)*∗X*(7) + *B*8*∗X*(2)*∗X*(3) + *B*9*∗X*(2)*∗X*(4) +  *B*10*∗X*(2)*∗X*(5) + *B*11*∗X*(2)*∗X*(6) + *B*12*∗X*(2)*∗X*(7) + *B*13*∗X*(3)*∗X*(4) + *B*14*∗X*(3)*∗X*(5) +  *B*15*∗X*(3)*∗X*(6) + *B*16*∗X*(3)*∗X*(7) + *B*17*∗X*(4)*∗X*(5) + *B*18*∗X*(4)*∗X*(6) + *B*19*∗X*(4)*∗X*(7) +  *B*20*∗X*(5)*∗X*(6) + *B*21*∗X*(5)*∗X*(7) + *B*22*∗X*(6)*∗X*(7) + *C*, where *Y* is a dependent variable of the flavonoid yield, *X*(1), *X*(2),…, *X*(7) are the seven independent variables associated with inoculum size, PH value, initial liquid volume, temperature, seed age, fermentation time, and rotation speed, respectively, and *A*, *B*, and *C* are real numbers.

Although the relationship between the data may not be linear, we can put squared term for a type of data into these data. If this term is useful it will be retained after linear regression analysis; otherwise, the data will be deleted.

In the regression analysis, it needs to focus on the values of *R*-squared and the significance of correlation coefficients for regulating the model. We use the regression analysis tools in the IBM SPSS, setting regression coefficients as estimated (*E*) and selecting the display model fit (*M*). Set the stepping method criteria as use of probability *F*, entry (*E*) as 0.5, and removal (*M*) as 0.10. After regression analysis, we can get the results as shown in Table 4.

It is obtained that significance = 0.006 < 0.05; that is, the regression results are obvious. *R*-squared value is 0.88, which means that the model is valid for fitting the 88% data. We get the statistical model: *Y* = 3662.278*∗x*(1) − 4263.361*∗*(*x*(1)^2^) + 11737.986*∗x*(2) − 999.556*∗x*(2)^2^ − 0.238*∗x*(3)^2^ + 3353.461 *∗* *x*(4) − 59.662*∗x*(4)^2^ − 420.854*∗x*(5) + 42.495*∗x*(5)^2^ + 966.796*∗x*(6) − 53.489*∗x*(6)^2^ + 27.213*∗x*(7)  − 0.234*∗x*(7)^2^ + 0.434*∗x*(3)*∗x*(7) − 86781.046.

### 3.2. Gene-Set Based Genetic Algorithm

**Figure 1:**
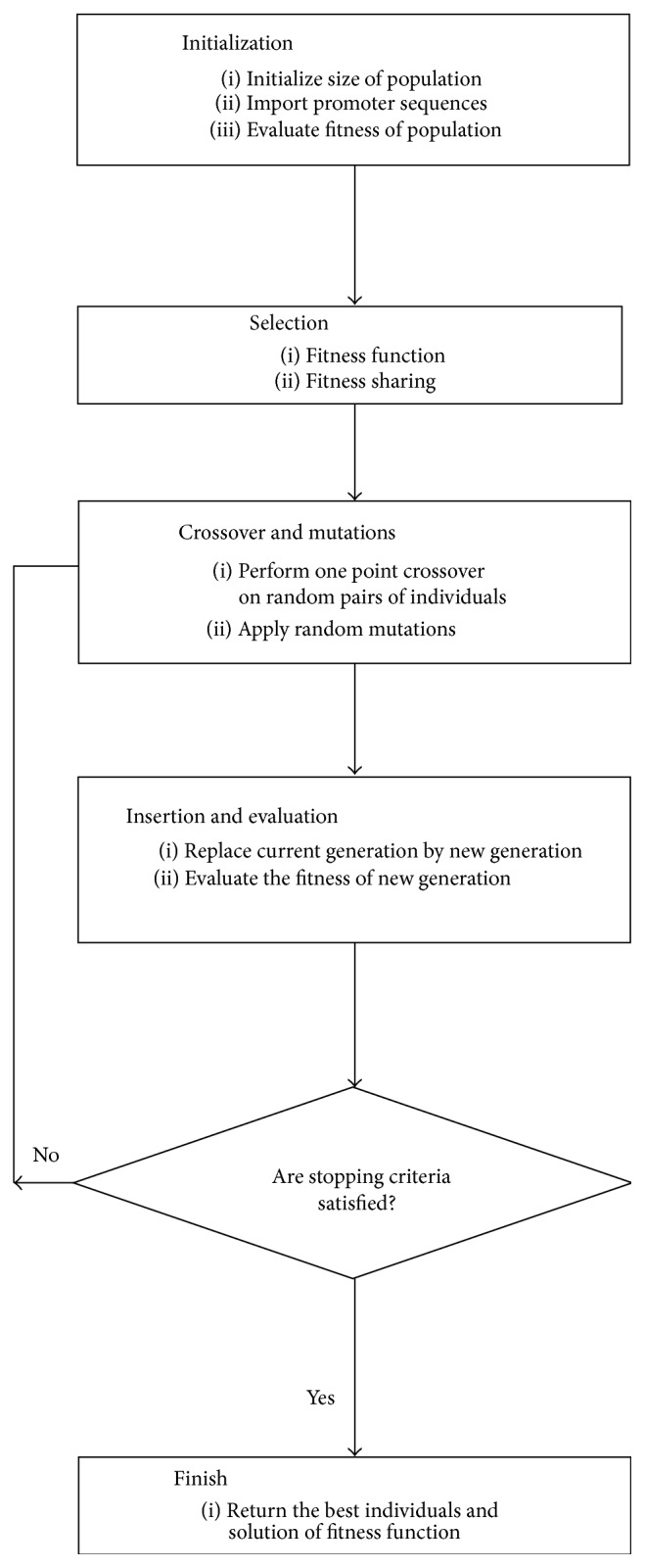
GA process.

Genetic algorithm (GA) was first proposed by J. Holland in 1975 [25, 26], whose general process is shown in Figure 1. In the mutation operation, if a short segment is selected in a mutation possibility and replaced by another segment, then the gene-set based GA is achieved [27].

In gene-set based GA, a chromosome is treated as a set of gene-sets, instead of a set of genes as in classical GAs. It starts with gene-sets of the largest size equal to half the chromosome length. It is most appropriate to genetics model because each gene-set represents a set of adjacent parameters of certain factor of the culture conditions.

It is noted that, in the selection, only the winning individuals from the population can be selected. Select operators are also known as reclaimed operator (reproduction operator), whose purpose is to optimize the selection of individuals (or solutions) to the next generation. Population can be updated by fitness ratio method and random sampling method to traverse, local selection. Cross operator refers to the part of the structure of the two parent individuals to generate new recombinant replacing individual operation. Variation is to make GA have local random search capability. When the GA crossover neighborhood is close to the optimal solution, the use of such a mutation operator of local random search capability can accelerate the convergence to the optimal solution.

The statistical model obtained by regression analysis is used as the fitness function here, and gene-set based GA is used to optimize the culture condition for maximizing the production of* Phellinus*. The data simulation is achieved by gatool in MATLAB. In the data experiments, we use a binary string composed of 7 segments to represent an individual in GA population, where each segment is associated with the value of one of the 7 parameters for the culture condition. Initial population size is 50, and cross rate is set to 0.8. Mutation rate is set to be 0.01, and selection method is roulette wheel selection. If the time is long enough then the GA process will halt by meeting the stopping conditions, such as generations limit or fitness limit.

**Figure 2:**
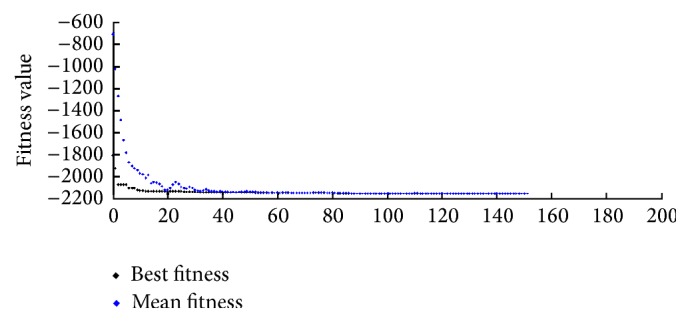
GA best fitness.

After 156 iterations the gene-set based GA process returns the best individual and shuts down the process in Figure 2.

After the regression analysis and GA process, an optimized culture condition is obtained, shown in Table 5.

The results obtained by our method have accordance with experimental experience in literature of* Phellinus* growth environmental studies. Specifically, the suitable environment is neutral acidic environment, about PH value 6. The appropriate temperature range is from 22°C to 28°C [10]. Seed age and fermentation time of species vary due to the strain [3, 28, 29]. These optimized values of the parameters have accordance with biological experimental results, which indicate that our method has a good predictability for culture conditions optimization.

## 4. Conclusion

In this work, 45 experiments are firstly operated for collecting data related to the production of* Phellinus* from* Phellinus* flavonoids. We use regression analysis method to create a mathematical model with the collected data, and then a gene-set based GA is proposed to optimize the culture condition, where the obtained mathematic model is used as fitness function for the evolution of individuals. In the comparison results, it is believed that PH value is credible and the temperature is also within the appropriate temperature range. Taking into account environmental factors in the laboratory, the temperature value we predicted is also reliable. The seed age and fermentation time predicted are 9, close to the original data 8. Data experimental results show that predicted optimal values of the parameters have accordance with biological experimental results, which indicate that our method has a good predictability for culture conditions optimization.

Neural-like computing models, such as artificial neural networks [30], spiking neural networks [31], and spiking neural P systems [32–34], have been successfully used in pattern recognition and engineering practice. It is of interest to use these neural-like computing models for optimizing culture conditions for* Phellinus* production. Our work would also guide for the “Precision Medicine” with personal SNP data [35] and other tasks in bioinformatics [21, 22].

## Figures and Tables

**Table 1:** Experiments with PH values ranging from 1 to 14 and initial volume ranges from 40 mL to 140 mL.

*Phellinus* production	PH	Temp.	Initial volume	Rotation speed	Including inoculum	Seed age	Fermentation time
45.929	1	28°C	100 mL	140	5%	8	8
35.077	2	28°C	100 mL	140	5%	8	8
45.654	3	28°C	100 mL	140	5%	8	8
534.39	4	28°C	100 mL	140	5%	8	8
702.81	5	28°C	100 mL	140	5%	8	8
1467.7	6	28°C	100 mL	140	5%	8	8
189.20	7	28°C	100 mL	140	5%	8	8
91.049	8	28°C	100 mL	140	5%	8	8
60.841	9	28°C	100 mL	140	5%	8	8
57.255	10	28°C	100 mL	140	5%	8	8
43.238	11	28°C	100 mL	140	5%	8	8
36.288	12	28°C	100 mL	140	5%	8	8
20.943	13	28°C	100 mL	140	5%	8	8
22.306	14	28°C	100 mL	140	5%	8	8

508.495	6	28°C	40 mL	140	10%	8	8
900.662	6	28°C	60 mL	140	10%	8	8
1273.594	6	28°C	80 mL	140	10%	8	8
1153.937	6	28°C	100 mL	140	10%	8	8
1123.330	6	28°C	120 mL	140	10%	8	8
1088.064	6	28°C	140 mL	140	10%	8	8

**Table 2:** Experiments with including inoculum ranging from 2% to 16% and temperature ranging from 25°C to 40°C.

*Phellinus* production	PH	Temp.	Initial volume	Rotation speed	Including inoculum	Seed age	Fermentation time
546.609	6	28°C	100 mL	140	2%	8	8
606.345	6	28°C	100 mL	140	4%	8	8
1320.794	6	28°C	100 mL	140	6%	8	8
1447.519	6	28°C	100 mL	140	8%	8	8
1841.729	6	28°C	100 mL	140	10%	8	8
1631.990	6	28°C	100 mL	140	12%	8	8
481.1172	6	28°C	100 mL	140	14%	8	8
449.5187	6	28°C	100 mL	140	16%	8	8

1145.669	6	25°C	40 mL	140	10%	8	8
1506.055	6	30°C	60 mL	140	10%	8	8
1374.982	6	35°C	80 mL	140	10%	8	8
875.341	6	40°C	100 mL	140	10%	8	8

**Table 3:** Experiments with fermentation time ranging from 1 to 12 hours.

*Phellinus* production	PH	Temp.	Initial volume	Rotation speed	Including inoculum	Seed age	Fermentation time
56.606	6	28°C	100 mL	150	2%	8	1
83.435	6	28°C	100 mL	150	4%	8	2
303.984	6	28°C	100 mL	150	6%	8	3
449.919	6	28°C	100 mL	150	8%	8	4
777.331	6	28°C	100 mL	150	10%	8	5
1103.987	6	28°C	100 mL	150	12%	8	6
1619.554	6	28°C	100 mL	150	14%	8	7
1597.995	6	28°C	100 mL	150	16%	8	8
1546.336	6	28°C	100 mL	150	10%	8	9
1502.487	6	28°C	100 mL	150	10%	8	10
1489.364	6	28°C	100 mL	150	10%	8	11
1465.664	6	28°C	100 mL	150	10%	8	12

**Table 4:** Regression analysis results.

	Sum of square	df	Mean square	*F*	*R*	*R*-squared	Standard error
Regression	3796787.42	14	249770.53	5.234	0.93	0.88	218.48
Residuals	47719.89	10	47719.54				
Sum	3973983.26	24					

**Table 5:** Optimized culture conditions.

Type	Experiment data	Computer data
Inoculum size	10%	12%
PH	6	5.8
Initial liquid volume	100 mL	100 mL
Temperature	28°C	28°C
Age	8	9
Fermentation time	8	9
Rotation speed	150	150
Flavonoid yield	2164.512	2150.128
